# Epidemiology of *CLOSTRIDIUM DIFFICILE* infection among active duty United States military personnel (1998-2010)

**DOI:** 10.1186/1471-2334-13-609

**Published:** 2013-12-28

**Authors:** Ramiro L Gutiérrez, Mark S Riddle, Chad K Porter

**Affiliations:** 1 Enteric Diseases Department, Naval Medical Research Center, 503 Robert Grant Ave, 20910, Silver Spring, Maryland, USA

**Keywords:** Clostridium difficile, C difficile, *Clostridium difficile* associated disease, Epidemiology, US military

## Abstract

**Background:**

*Clostridium difficile* associated disease (CDAD) has risen in incidence and the experience in the US military has not been described.

**Methods:**

We evaluated the U.S. military’s database and identified CDAD cases and demographic characteristics among affected military personnel from 1998 to 2010.

**Results:**

2,423 cases were identified. CDAD incidence was 13.2 cases (95% CI: 12.7-13.7) per 100 K p-yr and increased over study years. CA-CDAD and HA-CDAD incidence was 5.5 (95% CI: 5.2, 5.9) per 100 K p-y and 1.3 (95% CI: 1.2, 1.4) per 1,000 hospitalizations respectively. Females comprised a larger proportion of CA-CDAD than HA-CDAD (25.5% vs. 19.3%; p < 0.001) cases as did Air Force service (29% vs. 23.4%; p < 0.01). On multivariate analysis female gender, Coast Guard or Air Force service, and a married status was associated with CA-CDAD whereas Male gender and Marine Corps service were associated with HA-CDAD cases.

**Conclusions:**

CDAD has increased among military personnel, with female cases more likely to be community associated. Gender, marital status and branch of service had the strongest association with CDAD subtype. Further work is needed to evaluate the epidemiologic factors that have led to these increased rates in otherwise low-risk populations and associated sequelae.

## Background

*Clostridium difficile* associated disease (CDAD) usually manifests as an inflammatory, cytotoxin-mediated enteric disease with a wide spectrum of severity which includes asymptomatic carriage, acute or persistent diarrhea, and fulminant colitis with sepsis [1]. CDAD has increased in incidence and virulence in recent decades [1-4]. Recurrent disease and serious morbidity have also increased, especially among the elderly. Although the reasons for the increase are yet to be fully elucidated, emergence of hypervirulent strains, increased quinolone and other antibiotic use, as well as increased awareness and use of improved diagnostic tests have been implicated [1]. Novel risk factors such as proton pump inhibitor (PPI) and other medication use may also increase risk and be associated with these trends [5-8]. No longer a strict nosocomial illness, incidence among historically low-risk groups such as community dwellers, pregnant females and children represent important epidemiologic changes which underscore a need to better characterize the epidemiology of CDAD in younger cohorts [1].

US military personnel have full access to government paid medical care and are recognized to be a distinct population composed of younger and generally healthier individuals who also have unique exposures associated with worldwide military service. The US Department of Defense’s Armed Forces Health Surveillance Center (AFHSC) maintains comprehensive medical encounter databases on all service members as part of surveillance and public health efforts: the Defense Medical Surveillance System (DMSS) and the Defense Medical Encounter Database (DMED) [9]. The objective of our study was to use available military surveillance and medical encounter data to examine the epidemiology of CDAD among active duty personnel over the last decade. A cohort of all diagnosed CDAD cases was constructed and cross-sectional analysis performed.

### Methods

#### Description of the study population

*C. difficile* cases were identified from among active duty servicemembers from 1998 through 2010. Medical information and demographic data were obtained from inpatient and outpatient medical encounter and personnel databases by the Defense Medical Surveillance Center, The Armed Forces Health Surveillance Center, U.S. Department of Defense, Silver Spring, Maryland [inclusive dates:1998-2010; release date: March 2012]. All identifiable information was removed and subsequently provided to study investigators.

The study protocol was approved by the Naval Medical Research Center Institutional Review Board in compliance with all applicable Federal regulations governing the protection of human subjects. Datasets were de-identified and therefore a waiver of informed consent was granted by the Institutional Review Board.

CDAD cases were identified by extracting individual encounters with ICD-9 codes for clinician diagnosed CDAD (008.45). Setting of infection, community vs. healthcare-associated, was defined using modified Centers of Disease Control and Society of Healthcare Epidemiology of America (CDC/SHEA) case definitions [10]. Community acquired CDAD cases were individuals without inpatient medical encounters in the twelve-week period prior to CDAD diagnosis. Healthcare associated cases were those diagnosed while inpatients or who had been admitted in the twelve weeks prior to diagnosis. Clinical and demographic characteristics were collected from all cases.

### Analysis

Demographic comparisons between CDAD cases (CA- and HA-) were assessed using Chi-square test for categorical variables or Wilcoxon rank-sum (Mann-Whitney) test, as appropriate, for continuous variables lacking a normal distribution. Incidence rates and 95% confidence intervals were calculated using person-year denominator data for the study years for personnel in active duty service (CA) and hospitalizations (HA). Multivariate analysis of cross-sectional demographic and clinical variables associated with CA- vs. HA-CDAD was performed using log-binomial models to estimate prevalence ratios (PR). Covariates associated with CA- or HA-CDAD on univariate analyses were included in the final models using a stepwise selection approach with terms removed and added based on significance levels of 0.20 and 0.15 respectively.

Re-hospitalization rates were calculated by identifying inpatient encounters for C. difficile disease which occurred after 7 days and within 60 days from the original *C.difficil*e diagnosis.

All statistical analyses were performed using SAS v. 8.2 for Windows (SAS Institute, Cary, NC) and Stata version 12 (Stata Corp LP, College Station, TX) for Windows and statistical significance was evaluated using an alpha = 0.05.

## Results

### Study cohort and baseline characteristics

A total of 2,423 active duty service members were diagnosed with CDAD (CA-1,018; HA-1,405) over the study period for an overall incidence of 13.2 cases/100 K p-yr (95% CI: 12.7,13.7). The baseline characteristics of cases are shown in Table 1. Demographic variables closely paralleled the composition of the military services. Study subjects were predominantly young (median age 30), male (77.6%), and Caucasian (69.6%). The largest proportion of cases (41.1%) was from the Army while less than 5% were from the Coast Guard. Among the HA-CDAD cases diagnosed in outpatient clinics, 338 (24.0%) had a hospitalization in the 12-weeks prior to diagnosis.

**Table 1:** Demographics and descriptive epidemiology of U.S. military (CDAD) cases (1998-2010)

	***All cases***	***C. difficile *** **cases (CDAD) n (%) total = 2,423**		
	***2,423***	***CA = 1,018***	***HA = 1,405***	**P value**^**†**^
**Age yr. (Median; IQR)**	**30; 24, 39**	**31; 25, 39**	**30; 23, 39**	**0.1529**
**Gender**				
** Male**	**1,880 (77.6)**	**908 (74.5)**	**972 (80.7)**	**<0.001**
** Female**	**543 (22.4)**	**311 (25.5)**	**232 (19.3)**	
**Race**				
** White**	**1,686 (69.6)**	**853 (70)**	**833 (69.2)**	**0.263**
** Black**	**355 (14.7)**	**179 (14.7)**	**176 (14.6)**	
** Asian**	**39 (1.6)**	**21 (1.7)**	**18 (1.5)**	
** American Indian**	**26 (1.1)**	**19 (1.6)**	**7 (0.6)**	
** Hispanic**	**121 (5.0)**	**57 (4.7)**	**64 (5.3)**	
** Unknown/other**	**196 (8.1)**	**90 (7.4)**	**106 (8.8)**	
**Rank**				
** O6-O10**	**32 (1.3)**	**16 (1.3)**	**16 (1.3)**	**0.900**
** O1-05**	**360 (14.9)**	**185 (15.2)**	**175 (14.5)**	
** Warrant officer**	**34 (1.4)**	**16 (1.3)**	**18 (1.5)**	
** E5-E9**	**1,139 (47)**	**581 (47.7)**	**558 (46.4)**	
** E1-E4**	**858 (35.4)**	**421 (34.5)**	**437 (36.3)**	
**Education**				
** Graduate**	**346 (6.1)**	**98 (8.0)**	**80 (6.6)**	**0.001**
** Bachelor’s degree**	**675 (11.9)**	**164 (13.5)**	**153 (12.7)**	
** Some college**	**591 (10.5)**	**174 (14.3)**	**129 (10.7)**	
** High school**	**3,824 (67.6)**	**749 (61.4)**	**795 (66.0)**	
** <High school**	**42 (0.7)**	**4 (0.3)**	**19 (1.6)**	
** Unknown**	**176 (3.1)**	**30 (2.5)**	**28 (2.3)**	
**Marital status**				
** Married**	**3,160 (56)**	**753 (61.8)**	**686 (57.1)**	**0.006**
** Single never married**	**2,211 (39.1)**	**380 (31.2)**	**452 (37.6)**	
** Other**	**273 (4.8)**	**83 (6.8)**	**63 (5.2)**	
** Unknown**	**4 (0.1)**	**2 (0.2)**	**1 (0.1)**	
**Service branch**				
** Army**	**995 (41.1)**	**505 (41.4)**	**490 (40.7)**	**0.009**
** Air Force**	**642 (26.5)**	**354 (29.0)**	**288 (23.9)**	
** Coast Guard**	**73 (3.0)**	**36 (3.0)**	**37 (3.1)**	
** Marine corps**	**252 (10.4)**	**109 (8.9)**	**143 (11.9)**	
** Navy**	**461 (19.0)**	**215 (17.6)**	**246 (20.4)**	
**Follow up in days (Median duration)**	**677**	**814**	**608**	**<0.001**

†Wilcoxon Rank Sum (Mann-Whitney) test used for comparison of continuous variables (Age, Duration of Follow Up); All other categorical variables evaluated with Chi-Square test for comparison of frequencies by appropriate degrees of freedom. HA- vs. CA- comparisons.

Females constituted a larger proportion of the CA-CDAD cases than HA-CDAD cases (25.5% vs. 19.3%; p < 0.001). In terms of branch of service, the majority of total and HA-CDAD cases came from the Army, while the Navy and Coast Guard cases were evenly distributed among HA- and CA-CDAD cases, and CDAD was more common in Air Force members (29% vs. 23.5%; p < 0.001). Among Marines, HA-CDAD occurred more commonly (11.9% vs. 8.9%; p < 0.009). There were no significant differences in the age, military rank or race of the CDAD cases. The duration of follow-up (end of active duty service) was longer for those with CA-CDAD than those with HA-CDAD.

### Incidence of *Clostridium difficile*

#### Community acquired

The overall incidence (Table 2) of CA-CDAD was 5.5 cases per 100 K p-yr (95% CI: 5.2, 5.9). Among the branch of service, rates of CA-CDAD were highest in the Coast Guard, Army and Air Force with lower rates in the Navy and Marine Corps. By age category and gender, rates of CA-CDAD were significantly higher among females (10.4 vs. 4.6 cases per 100 K p-yr) and increased significantly by age with the highest rates seen among those over 40 with 12.0 cases per 100 K p-yr (95% CI: 10.6, 13.7).

**Table 2:** **Incidence of HA and CA-*Clostridium difficile *** **infection over the study period among U.S. military personnel (1998-2010)**

	***CA-CDAD***		***HA-CDAD***	
***Category***	***Rate (Cases per 100 K p-yr)***	***95% CI***	***Rate (Cases per 1 K hospitalizations)***	***95% CI***
**Overall incidence**	**5.5**	**5.2-5.9**	**1.3**	**1.2-1.4**
**Branch of service**				
**Army**	**6.3**	**5.7-6.9**	**1.2**	**1.1-1.3**
**Navy**	**4.0**	**3.5-4.7**	**1.2**	**1.1-1.4**
**Air Force**	**7.2**	**6.4-8.0**	**2.7**	**2.5-2.9**
**Marines**	**2.7**	**2.1-3.5**	**2.2**	**1.9-2.5**
**Coast Guard**	**8.7**	**6.4-11.7**	**Not available**	
**Age**				
**<20**	**1.8**	**1.3-2.7**	**0.5**	**0.4-0.7**
**20-24**	**3.8**	**3.3-4.3**	**1.1**	**1.0-1.3**
**25-29**	**5.6**	**4.9-6.4**	**1.4**	**1.2-1.6**
**30-34**	**6.1**	**5.3-7.1**	**1.7**	**1.5-2.0**
**35-39**	**6.4**	**5.5-7.5**	**1.7**	**1.5-1.9**
**> = 40**	**12.0**	**10.6-13.7**	**1.4**	**1.3-1.6**
**Race**				
**Black**	**4.6**	**3.9-5.3**	**0.9**	**0.7-1.0**
**White**	**6.0**	**5.5-6.4**	**1.3**	**1.2-1.4**
**Native American**	**6.0**	**3.5-10.2**	**Not available**	**-**
**Hispanic**	**2.3**	**1.7-3.2**	**Not available**	**-**
**Asian**	**2.1**	**1.3-3.5**	**Not available**	**-**
**Other/unk**	**8.0**	**6.2-10**	**0.8**	**0.7-1.0**
**Gender**				
**Male**	**4.6**	**4.3-5.0**	**1.7**	**1.6-1.8**
**Female**	**10.4**	**9.2-11.7**	**0.6**	**0.5-0.7**

### Hospital acquired/associated

During the study period, the overall incidence of HA-CDAD was1.3 (95% CI: 1.2, 1.4) cases per 1,000 hospitalizations (Table 2). The Air Force and Marine Corps had somewhat higher rates than the other services, excluding the Coast Guard from which detailed hospitalization rates were unavailable. By age category, rates of HA-CDAD were largely similar for the different age groups in our study population without a clear increased incidence among the oldest service members. By gender, rates of HA-CDAD were reversed and higher for males (1.7 cases per 1 K hospitalizations; 95% CI: 1.6, 1.8) than for females (0.6 cases per 1 K hospitalizations; 95% CI: 0.5, 0.7).

### CDAD incidence trends

**Figure 1:**
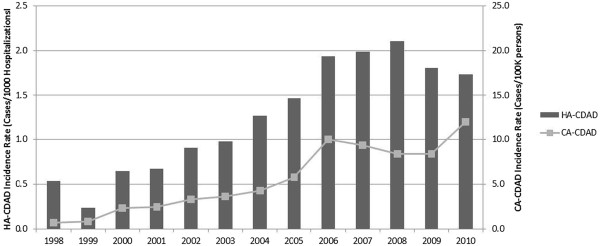
**CDAD burden among US military personnel, from 1998 to 2010.** Incidence of HA-CDAD is expressed per 1,000 admissions (left Y-axis); incidence of CA-CDAD is expressed per 100,000 person-years (right Y-axis).

**Figure 2:**
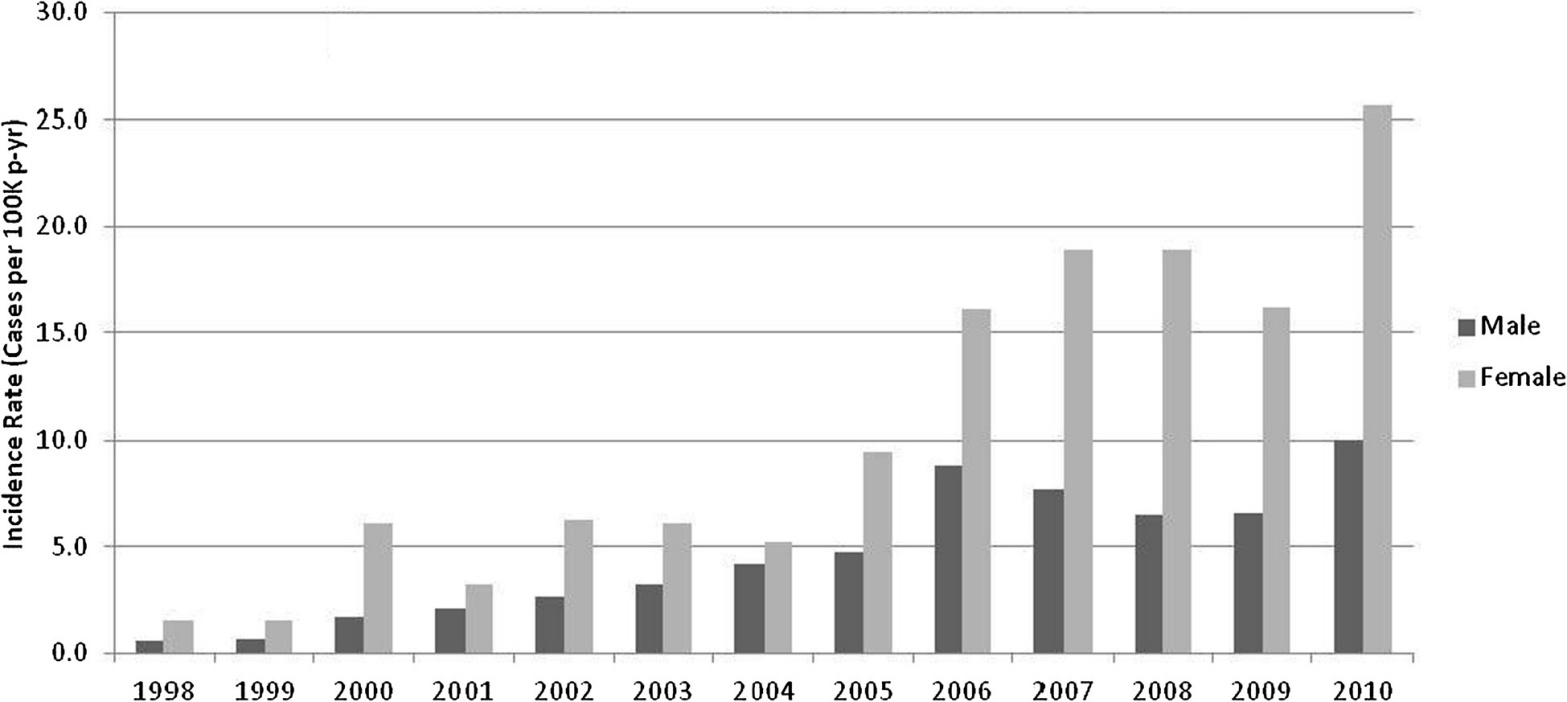
**CA-CDAD burden, by gender, among US military personnel, from 1998 to 2010.** Incidence is expressed per 100,000 person-years.

**Figure 3:**
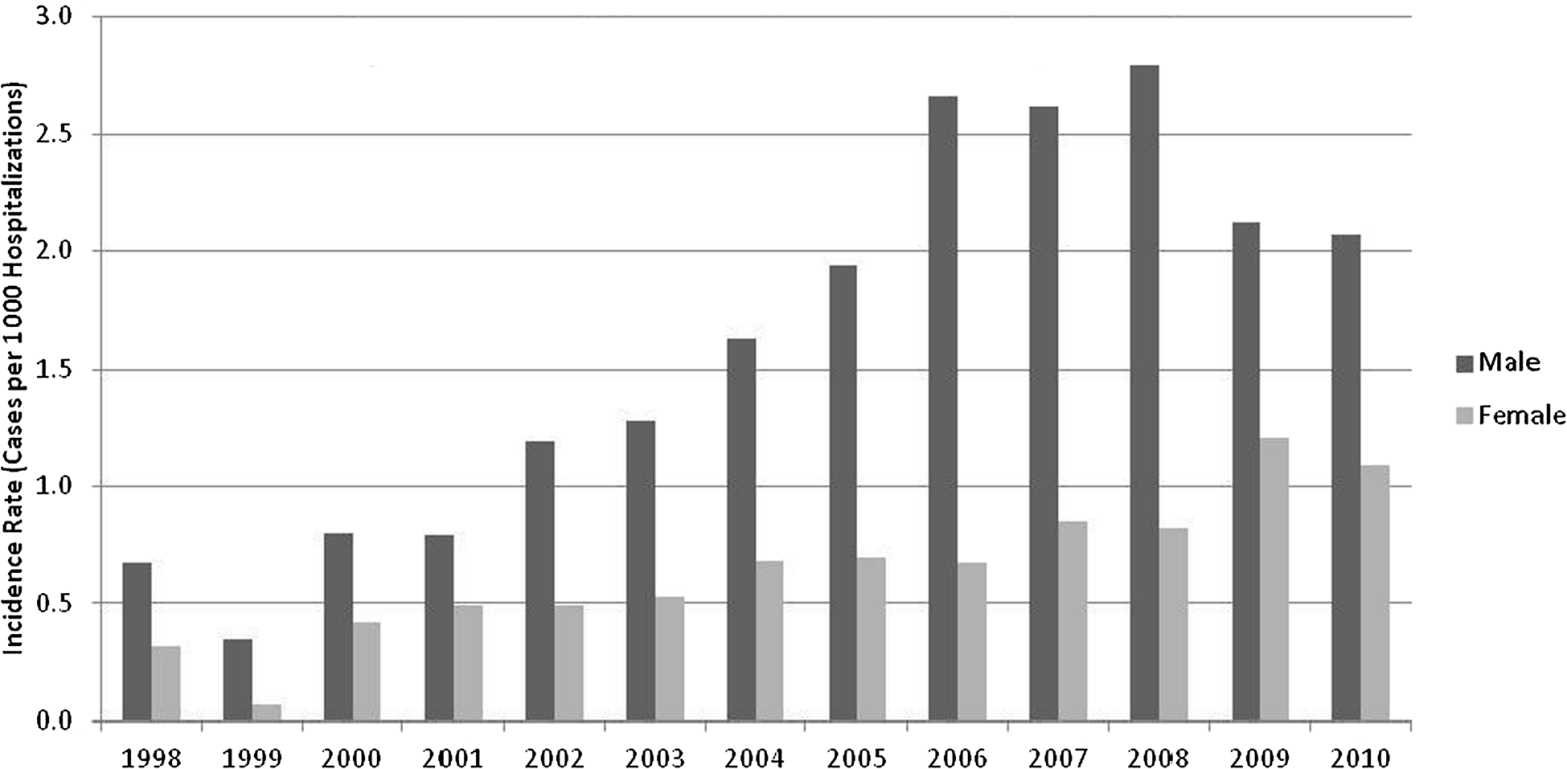
**HA-CDAD burden, by gender, among US military personnel, from 1998 to 2010.** Incidence is expressed per 1,000 hospitalizations.

CDAD incidence increased notably over the study period. While an overall estimated incidence of less than five cases per 100 K p-yr was seen in 1998-1999, by 2008 CDAD incidence had increased to greater than 20 cases per 100 K p-yr. The rise occurred in both CA- and HA- cases (Figure 1). Incidence trends by gender also revealed a rise for both men and women over the study period (Figures 2 and 3). Branch of service contribution to the CDAD cases changed somewhat over the study period with a larger relative incidence for Army in the later years (38-40% of all cases), and decreased for proportions from the Air Force (data not shown).

### Prior hospitalizations

The hospitalization history of all CDAD cases was evaluated. Among the 1,405 HA-CDAD cases, 1,066 (76%) were inpatients at the baseline visit and for 728 of those (51.8% of all HA-CDAD cases) the baseline visit represented their only hospitalization on record. Among all HA-CDAD cases, if a prior admission was present it most often occurred in the 1 month prior to the CDAD diagnosis. Specifically, for outpatients and inpatients at the baseline visit, the median number of days from the last admission was 20 days (IQR: 35, 8) and 18 days (IQR: 38, 7) respectively (data not shown).

### Multivariate analysis and prevalence ratio

Table 3 illustrates prevalence ratios associated with demographic variables for CA-CDAD vs. HA-CDAD after adjustment for potential confounders. In the models, female gender and Coast Guard service was associated with CA-CDAD after adjustment for potential confounders identified during univariate analysis.

**Table 3:** Multivariate prevalence ratios for CA-CDAD (vs. HA-CDAD) among U.S. military personnel

***Category-covariates***	***Prevalence ratio***	***95% CI***	***p-value***
**CA-CDAD**			
**Female**	**1.28**	**1.15-1.42**	**<0.001**
**Married**	**1.16**	**1.04-1.29**	**0.0071**
**Air Force**	**1.14**	**1.03-1.27**	**0.0121**
**Marines**	**0.68**	**0.54-0.85**	**0.008**
**Coast Guard**	**1.55**	**1.27-1.89**	**<0.001**

### Duration of C. difficile disease and readmissions

Among all CDAD cases, 1,451 (60%) subjects had subsequent CDAD-related medical encounters after the initial visit and of these, 582 (40%) had two or more visits during the follow up period. For a majority of both CA- and HA-CDAD cases, the last CDAD-related code occurred within a year after diagnosis. Of the 1,018 subjects identified as CA-CDAD cases, 519 had subsequent CDAD coded visits with the majority occurring within a month of the original visit, and 43 (4.2%) and 64 (6.3%) having a CDAD visit coded after 8 weeks and 4 weeks respectively. Among the 1,405 HA-CDAD cases, 932 had subsequent CDAD coded visits the majority of which also occurred within a month of the original diagnosis. For HA-CDAD cases, 68 (4.9%) and 140 (9.9%) had CDAD visits after 8 and 4 weeks, respectively, from the original diagnosis (Table 4).

**Table 4:** Recurrence/relapse and re-hospitalization of CA-CDAD vs. HA-CDAD cases

***CDAD visits afte:r index date***	***N***	***%***
**CA-CDAD (n = 1,108)**		
**Visits after 2 wks**	**91**	**8.9**
**Visits after 4 wks**	**64**	**6.3**
**Visits after 8 wks**	**43**	**4.2**
**Hospitalization within 2mo.**	**4**	**0.3**
**HA-CDAD (n = 1,405)**		
**Visits after 2 wks**	**228**	**16.2**
**Visits after 4 wks**	**140**	**9.9**
**Visits after 8 wks**	**68**	**4.8**
**Hospitalization within 2mo.**	**96**	**6.8**

In terms of re-hospitalization rates for CDAD cases. Among all the exposed (n = 2,421), 100 (4.3%) subjects had an inpatient encounter within 2 months for CDAD. Ninety-six of the admissions were among cases previously classified as HA-CDAD, whereas only 4 cases were among the previously classified as CA-CDAD (Table 4).

## Discussion

In this generally healthy population of young, active-duty US military personnel, we noted a marked rise in the incidence of CDAD both in terms of hospital and community associated disease. The overall CDAD incidence was 13.2 cases per 100 K p-y, but was notably skewed towards higher rates in the latter years of our study period with 10-fold and 4-fold increases in incidence for CA-CDAD and HA-CDAD respectively. A rise in CDAD incidence has been well documented in many populations and is associated with increased morbidity and mortality among the elderly, infection of previously low-risk groups, and emergence of novel risk factors such as PPI use [1-3,10-13]. A stabilization and slight rate decrease after 2009 is seen among our HA-CDAD cases, a trend also noted in other published reports [12,14]. Among CA-CDAD cases, and for females, rates for the last study year were notably higher than in prior years but care must be taken when interpreting this observation. Implementation of PCR based testing for CDAD became more prevalent among DoD medical facilities in this period may have contributed to this observation but also our calculated rates for females may have been subject to more variability as a minority of US military personnel are female.

Our study was not designed to evaluate specific risk factors associated with CDAD, yet some notable associations were observed in terms of the demographic variables evaluated. Similar to prior reports, women were more frequently associated with the CA-CDAD cases [11,15] with a gender specific rate markedly higher than males in our cohort during the overall study period. Specific determinants associated with this observation cannot be ascertained from our data but differential rates of exposure to risk factors for CDAD acquisition or diagnosis (antibiotic use, healthcare system exposure, host factors, etc.) may be contributory. Evaluation of additional risk factors for *C. difficile* acquisition and development of disease in the community setting is needed and may further validate and explain the gender specific differences in incidence and prevalence which have been observed.

Similarly, in our population, racial distribution of cases of CDAD was not uniform and those described as Black, Caucasian and Native American had higher rates than Hispanic and Asian groups. Published data from other populations have differed and revealed higher rates of CDAD and associated morbidity in persons of Caucasian ancestry while other reports have demonstrated lower incidence rates among Asian and other minority groups [16]. More study is needed to further elucidate any genetic or environmental factors to explain this association in the military and other populations.

Our study found that specific CDAD rates by each branch of military service also demonstrated some differences. The Coast Guard, Air Force and Army shared similar rates of CA-CDAD which were higher than those seen in the Navy and Marine Corps. On the multivariate models, Coast Guard or Air Force service was independently associated with CA-CDAD in our population and suggests that in addition to basic demographics, other factors may contribute to the different rates seen in our service populations.

Recurrence of disease due to relapse or re-infection is increasingly a significant management challenge. Resistance to antibiotics, novel virulence determinants, and ongoing host factors (need for broad spectrum antibiotic therapy, immune compromise, continued exposure to pathogen) all appear to contribute to recurrent disease [17,18]. In our population, the majority of CA and HA cases did not have continued visits for CDAD after 4 and 8 weeks. For those with CDAD visits after those time points, the CDAD code was in the primary or secondary position and suggestive of relapse or re-infection. The highest rate was among hospital associated cases for which 6.3% had CDAD visits 8 weeks after the primary diagnosis. Reported recurrence rates from civilian cohorts are generally higher and range from 20-30% for both community and hospital associated cases [11,17-19]. In our population, the low rates of CDAD specific visits after 4 and 8 weeks is notable and suggest a lower rate of relapse or re-infection. Rates of hospitalization for CDAD in our cohort was more common among HA-CDAD cases, where 96 (6.8%) individuals were readmitted within 2 months with a CDAD diagnosis compared to only 4 (0.3%) of the CA-CDAD cases. Our study relied on ICD-9 codes to identify cases and poses a limitation in the ability to distinguish recurrence and relapse but our conservative approach makes it unlikely rates are significantly higher than estimated.

Our study has several strengths and limitations. Access to the large, worldwide deployed, US military population allowed us to select and follow a large number of CDAD cases from all services and a variety of clinical settings but for which access to care, testing and therapy is relatively uniform. This study represents the most comprehensive and extensive review of CDAD in the military population and reveals the epidemiology of CDAD largely has paralleled the trends seen in other civilian and older populations. A main limitation of our study is the medical encounter database nature of its design and reliance on ICD-9 coding which may increase the risk for misclassification. However, recently published validation studies in inpatient populations have noted a single CDAD ICD-9CM code has a high specificity for CDAD disease (Sp. 99.7%, Sen. 78%) and may therefore be adequate tools for surveillance [20]. One concern raised by these validation studies that may apply to our work is the observation that there is risk for misclassifying of CA-CDAD cases that are admitted to the hospital as being as HA-CDAD. This is a potential bias may have affected our results since we were unable to ensure that cases of CDAD less than 48 hr after admission were classified as community-acquired [13,20]. A lack of access to specific medical procedure and medication history precluded the ability to examine other risk factors for development of CDAD or adjustment by those potential confounders.

Multiple factors, including period effects, may have influenced the changes in CDAD rates seen in our study. Military specific factors, such as demographic transitions in the military population, and the effect of operational requirements with attendant changes in overall exposure to combat deployments may have influenced rates in our population during this time period but it is likely that most of these effects would have been limited. Interestingly, service in the Army and Marine Corps, younger, an enlisted populations are more heavily involved in combat operations, and were also more likely to be associated with HA-CDAD. In addition, other risk factors and secular trends, which have been documented from other populations (i.e. quinolone and proton-pump inhibitor use, availability of molecular diagnostics, increased provider awareness, surveillance bias), may also have influenced the rates and increases seen in our population.

### Conclusions

In summary, in this retrospective study of the generally younger, active duty U.S. military population, our data documents a notable rise in incidence of both CA-CDAD and HA-CDAD, similar to what has been seen in other populations. Of note, we documented a female association with CA-CDAD which persisted when controlling for service branch and low rates of recurrence or re-infection after the index visit. Incidence and risk factors for CDAD among military personnel likely differ from those of the general population, and our findings regarding the incidence and patterns of CDAD need further study.

## Competing interests

None of the authors have a conflict of interest or a financial disclosure related to this work which was performed as part of official duties.

## Authors’ contributions

RLG, CKP and MKR participated in the data analysis and drafting of the manuscript. All authors read and approved the final manuscript.

## Pre-publication history

The pre-publication history for this paper can be accessed here:

http://www.biomedcentral.com/1471-2334/13/609/prepub

## Summary

A review of the U.S. military reveals a decade with notable increases in CDAD incidence. Among these ‘healthy-workers’ both HA- and CA- show these trends. Demographic and service related variables were significantly associated with HA- and CA-CDAD.

## Copyright statement

The authors are military service members (or employee of the U.S. Government). This work was prepared as part of official duties. Title 17 U.S.C. §105 provides that ‘Copyright protection under this title is not available for any work of the United States Government.’ Title 17 U.S.C. §101 defines a U.S. Government work as a work prepared by a military service member or employee of the U.S. Government as part of that person’s official duties.

## Meetings/prior presentations

Limited portions of preliminary data used in this work were presented as a podium presentation at the Digestive Diseases Week conference 2012, San Diego CA.
